# The National Insurance Bill

**Published:** 1911-06-24

**Authors:** 


### THE NATIONAL INSURANCE BILL.

The list of proposed amendments of the National Insurance Bill continues to grow, and in the House of Commons questions relating to the Bill are asked daily. Dr. Addison has put down a series of amendments which he will move when Clause 14 is reached in Committee. The effect of these, if carried, would be to place the arrangements for medical service in the hands of local health committees, such arrangements to be subject to the approval of the Insurance Commissioners, and to allow insurance patients practically free choice of medical attendants. It is understood that the Chancellor of the Exchequer regards these amendments favourably, but he anticipates strong opposition from the Friendly Societies. An amendment which has been put down in the name of Mr. Barton provides that the rate of medical remuneration shall be so calculated as "to produce an amount equal to that which would be produced by private practice at the rates general in the district." One of the questions Mr. Lloyd George was called upon to answer arose out of his statement that since the insurance system came into force in Germany private benefactions to the hospitals have doubled, and that the experience of the only other country in which a scheme of national insurance has been introduced shows that the effect of insurance is not to diminish but to increase the subscriptions which are made to hospitals. When asked by Mr. Montague Barlow to state the increase of such benefactions and subscriptions in Germany subsequent to 1880, and also the increase to similar institutions in this country since that time, the Chancellor said it was impossible to give the comparative return desired, but he added that the German Government state that during the twenty-four years—1882 to 1906—the number of public and of private hospitals with more than ten beds increased from 2,024 to 3,801, and the number of beds from 83,005 to 222,687. While the population increased during the period named by 35 per cent., the number of hospitals increased by 88 per cent., and the number of beds by 168 per cent.

Another question related to soldiers in hospitals, Major Henderson having asked whether the 9d. per diem will be deducted from a soldier's pay while in hospital, in view of the fact that he will be a contributor to the insurance fund, Mr. Lloyd George replied that the reduced contributions payable by and in respect of a soldier while in service are needed to provide for the benefits due to him as an insured person after leaving the service. In any case, the position of a soldier as a contributor would not of itself justify his being treated more favourably in the matter of pay during sickness than a civilian insured in the ordinary way through an approved society. Asked by Mr. James Thomas whether medical assistance could be provided for old folks now in receipt of old-age pensions, the Chancellor said such an arrangement would impose a serious charge on the Exchequer, and it would not be consistent with the contributory character of the scheme to provide benefits at a nominal charge. Replying to Mr. Clynes, who asked whether, in fixing the medical expenditure at 6s. per head of insured persons, information had been obtained from the Post Office, police authorities, and similar public bodies as to how much they pay per head on their employees for medical attendance, Mr. Lloyd George said that a comparison of the scales of payment in the cases mentioned would be misleading. Notice has been given of several other questions affecting the medical and pharmaceutical services under the proposed scheme, and, in fact, the Bill is likely to be the main topic of interest during the remainder of the session.

Mr. Lloyd George, in a letter addressed to Sir Donald MacAlister, has replied to the various recommendations made by the General Medical Council. In this communication he says it is his present intention that a medical man shall have a seat upon the Insurance Commission and that the profession will be represented on the Advisory Committee and on the local health committees. He points out that the Bill contains provision for the transfer of medical attendance to the local health committees, but at this stage he does not think it possible to propose that societies should be compelled to hand over medical attendance to these committees, although he agrees that it is desirable. As to the "free choice of doctor," the Chancellor says there must be some limit to this freedom of choice, and there must be some method of securing a panel of practitioners which would prevent work being done by members of the profession who had shown themselves unfit for the performance of a very responsible public duty. With reference to the recommendation of the Council "that in respect of the grant of subscriptions and donations to hospitals and other charitable institutions contemplated in Clause 17 of the Bill, the governing conditions should be assimilated to those set forth in Clause 15 (1) with regard to the administration of sanatorium benefit," the Chancellor points out that the conditions in Clause 15 for sanatorium treatment make the approval of the Local Government Board necessary for the sanatoria to which the payments for treatment are made from the Insurance Fund. It is contemplated that this provision will carry with it a certain amount of inspection and control. He is not sure whether the Council fully realised this, and confesses that he should have hesitated to propose that public inspection and control should be extended to the voluntary hospitals which receive money from approved societies. He agrees that it will be necessary to include in the Bill the provision that prescriptions should be dispensed by duly qualified persons. It is not proposed to make provision in the Bill for institutional treatment and operations. Sickness benefit cannot be given to an insured woman as well as maternity benefit, but he will consider the suggestion that in cases attended by a midwife the local health committee shall be empowered to pay for the attendance of a medical practitioner should such attendance be called by the midwife.

